# Doctor Thomas Dimsdale, and Smallpox in Russia

**Published:** 1984-01

**Authors:** John Griffiths


### Doctor Thomas Dimsdale, and Smallpox in Russia.

### The Variolation of the Empress Catherine the Great

**John Griffiths**

Thomas Dimsdale came of a family many of whose members had been doctors, and studied at St. Thomas' Hospital, London until 1734. He settled at Hertford, as a surgeon, and married Mary Brassey whose father was an eminent banker, and a Member of Parliament. She died childless 10 years later, Thomas feeling her loss severely: to assist him to assuage his grief, he served gratuitously with the army, commanded by the Duke of Cumberland, which was advancing to oppose the Young Pretender: he served as an army surgeon until the surrender of Carlisle to Cumberland on 30th December 1745, when, after receiving the Duke's thanks, he returned to his practice in Hertford. In 1746 he married again a relative of his first wife, who brought him a large fortune: with this and an inheritance from a relative of his own he retired from medical practice, assuming the life and duties of a country gentleman, and devoting himself to the procreation and nurture of a family of ten children, seven of whom survived. These activities were however insufficient to absorb his energies: and in 1761 he again started in medical practice, taking an M.D. degree at the University of Aberdeen, and being admitted Extra-Licentiate of the Royal College of Physicians. Thomas had always been especially interested in smallpox inoculation: and he became acquainted with the work in this field of a family named Sutton, who despite the absence of any formal medical qualifications had achieved remarkable success with their techniques. These consisted of a 2–3-week preparatory diet and drug preparation followed by puncture of the skin with a lancet dipped, to use their own description, "in the smallest possible quantity of the unripe crude or watery matter from the pustules" from a patient suffering from the disease. The incision, one in each arm, was minute: it was not to exceed one eighth of an inch in length nor one sixteenth of an inch in depth: and the Suttons had started, and were operating with outstanding success an Inoculation House at Ingatestone in Essex, where patients became so numerous that the local village was often unable to accommodate them. They inoculated about 17,000 persons, with only five or six fatalities. Thomas Dimsdale studied their methods, and adopted them with slight modifications: and in 1767 published his first treatise on the subject, entitled "The Present Method of Inoculating for the Small Pox", a pamphlet of 160 pages, in which he acknowledges the pioneering work of the Suttons, albeit with the condescension of the highly qualified to the unlettered expressed in the observation that their success was "the more marvellous as the operators were chiefly such as by report could lay but little claim to medical education" and that he had studied their work "knowing that improvements which would do honour to the most elevated human understandings are sometimes stumbled upon by men of more confined abilities". He does not mention the Suttons by name, or indicate their locality. The success of Thomas' book was immediate, four editions being demanded in the year of its publication, and the seventh edition in 1779: his reputation was established, and people of all classes of society flocked to Hertford for inoculation.

It is said that in one year in the second half of the eighteenth century, two million people died of smallpox in Russia: that most remarkable Queen, Catherine the Great, Empress of All the Russias, had concerned herself anxiously with this terrible state of affairs. The Russian monarchs had always shown a strong predilection for British doctors: ever since 1557 when Robert Standish, a Cambridge M.D. had emigrated to Russia as physician to Ivan the Terrible, British medical men of eminence had held places at the Russian Court. It is likely that the reputation of the Suttons had reached Catherine's ears: a tragic death from smallpox of a member of the highest Russian nobility was the catalyst for action: and the Russian ambassador to the Court of St. James was instructed to secure the services at St. Petersburg of a physician with an established reputation in the specialty. The ambassador was at the time a patient of Doctor John Fothergill, who was himself a friend of Thomas Dimsdale: and when the ambassador sought the assistance of Fothergill, the latter at once recommended Thomas. A courier was dispatched to Hertford: and Thomas met the ambassador in

Paper read to the Bristol Portfolio Society on 18th November 1983.

London, but despite all the blandishments and inducements in the ambassador's armoury, Thomas declined the task. He was 56, a rich man, and deeply attached to his large family: and he had formidable commitments to his chosen interest in England: but he offered to find a substitute. While he was trying to do this, the ambassador again sought him to tell him that an officer of distinction, employed only on extraordinary occasions, had arrived from St. Petersburg, having completed the journey in the remarkable time of 16 days, such was his urgency: and that this emissary was personally charged by the Empress and the Grand Duke to reinforce the assault on Thomas. This time Thomas succumbed: and leaving the question of his remuneration "Entirely to the gracious pleasure of her Imperial Majesty" he made instant preparations to travel to St. Petersburg, receiving £1000 for his expenses from the Russian ambassador. He took with him his son Nathaniel, then a medical student at Edinburgh, but thoroughly familiar with his father's techniques. They set off on 28th July 1768, travelling rapidly and comfortably by chaise, with an escort, by way of Berlin, Dantzig and Riga, covering the distance from Amsterdam to St. Petersburg in exactly 1 month. A fine house was being prepared for them in Millionaire Street, but it was not ready when they arrived: so luxurious accommodation elsewhere, but close to the palace, with an elegant carriage were placed at their disposal, with every other adjunct conducive to their health, enjoyment and comfort. On the second day after their arrival, Thomas was received by Count Panin, the prime minister, with the utmost graciousness: and on the following day he and Nathaniel were presented to the Grand Duke Paul. On the next day Thomas was received by the Empress, in the presence of Count Panin, and Baron Cherkassoff, the President of the College of Medicine in St. Petersburg, who had been educated at Cambridge, and who spoke English perfectly: and he dined with the Empress that evening, being immensely impressed, not only by her charm and graciousness, but by the penetration and propriety of the questions which she asked him about inoculation. For some while Thomas and Nathaniel, now installed in a splendid mansion guarded by soldiers, devoted themselves to social and recreational activities, in which they received distinguished attention from the Court: but in due course Count Panin told Thomas that "To your skill and integrity will probably be submitted no less than the precious lives of two of the greatest personages in the world." Thomas comments: "Many corroding cares disturb me, and embitter all this greatness which I am not able to enjoy." Shortly afterwards Thomas was summoned to a second private interview with the Empress, who told him that she wished to be inoculated as soon as possible: Thomas requested the assistance of the court physicians, but the Empress refused, expressing complete confidence in him and pointing out that the court physicians were devoid of experience in inoculation, and therefore could give no help, and might be an embarrassment. Thomas then suggested that several persons of like age, sex and constitution to those of the Empress should be inoculated first, by way of a trial, but although the Empress declined even this precaution, it was in fact taken, as preparations were then being made for the establishment of an inoculation hospital under Doctor Schulenius, a physician of Livonia who had performed several successful inoculations in that province: and, under Thomas' general supervision, and with the aid of an assistant, inoculations started there, Nathaniel treating two 14-year-old boys with infected material taken from the child of a peasant whose smallpox was then approaching a crisis. One of these cases gave Thomas grave anxiety, to such an extent that the Empress noticed a change in his demeanour and enquired its cause. Notwithstanding a full and detailed disclosure to the Empress of the patient's unfavourable response, she continued to express complete confidence in Thomas, saying "I make no doubt with the blessing of God, he will be carried safe through his complaint and all will end well: I am satisfied with your conduct, and you may depend upon my protection and support: and whatever may be the event with this boy, it shall not alter my resolution provided you remain in the good opinion of my being inoculated." The Empress could not have afforded a more striking proof of her courage and dedication to the great cause which she was determined to promote. Both boys recovered: four more boys, and a girl were inoculated: in spite of some apparent imperfections in the process, the evidence indicated success in general: and the Empress would wait no longer. In October 1768, she started the preparatory diet and medicinal treatment: and three healthy children were inoculated to provide the infected material. One of these children, whose inoculation had reached the appropriate stage, was wrapped in a quilt while asleep, and taken by Thomas and Nathaniel to the palace: the Empress, in the presence only of Baron Cherkassoff, was quickly inoculated by one puncture in each arm: and the child donor Markoff was returned to the hospital, where he made a perfect recovery and was later ennobled for his services: his titular designation of nobility was Ospiennyi, "Ospa" being the Russian word for smallpox and his crest an arm bearing a rose in the hand, and an inoculation incision. The whole procedure had taken place at night, and in secret: and the next day the Empress moved to her summer palace, where she was joined by Thomas. Thomas records "A pretence has been found for her going to this palace, and the inoculation was not known till the fifth day: she has had the smallpox in the most desirable manner: a moderate number of pustules, and complete maturation, which now, thank God, is over, and I find an inexpressible load of concern removed from my breast." A little later the Grand Duke was inoculated with equal success: a great thanksgiving service was attended by the Empress, the Grand Duke, and many nobles at the Court chapel, during which the Empress announced that she had created Thomas a Baron of the Empire, a Councillor of State, and his appointment as Body Physician to the Empress. He was given a present of £10,000, £2000 for his expenses and a life annuity of £500, payable in England: and an augmentation of his arms in the form of a wing of the Russian eagle, on a gold shield, with helm and baronial coronet. Nathaniel was also created a Baron of the Empire.

Thomas and Nathaniel remained at St. Petersburg for another 4 months: they inoculated about 150 of the most distinguished nobility, and established an inoculation hospital in Moscow. They were loaded with valuable gifts, and were entertained lavishly at banquets, balls and shooting parties. They were about to return to England, when the Empress became seriously ill with pleurisy: Thomas was given exclusive charge of the patient: and remained until she was fully restored. On his return to England he was elected a Fellow of the Royal Society: he became a partner in a banking business in the City and Member of Parliament for Hertford: and paid a second visit to Russia in 1781 to inoculate the Princes Alexander and Constantine. He maintained his interest in inoculation, publishing the results of his experiences from time to time, and corresponding, sometimes in controversial terms, with his colleagues. Ironically he lived to see the death blow given to smallpox inoculation by the publication in 1798 of Jenner's work on vaccination, the introduction of which ended the earlier practice. He died at Hertford on 30th December 1800 in his 89th year.

### REFERENCES

DIMSDALE, Thos. (1767) The present method of inoculating for the Small Pox. London W. Owen

BISHOP W. J. (1932) Thos. Dimsdale, M.D., F.R.S. (1712–1800) and the inoculation of Catherine the Great of Russia. *Ann. Med. Hist. New Series. Vol. iv, 321–338.*

CRONIN, Vincent (1978) Catherine Empress of all the Russias. Collins, pp. 167–169, 323.

